# Urinary shedding of pathogenic *Leptospira* in stray dogs and cats, Algiers: A prospective study

**DOI:** 10.1371/journal.pone.0197068

**Published:** 2018-05-16

**Authors:** Sara Zaidi, Amar Bouam, Amina Bessas, Djamila Hezil, Hicham Ghaoui, Khatima Ait-Oudhia, Michel Drancourt, Idir Bitam

**Affiliations:** 1 Ecole Nationale Supérieure Vétérinaire, Alger, Algérie; 2 Aix Marseille Univ, IRD, MEPHI, IHU Méditerranée Infection, Marseille, France; 3 Ecole Supérieure en Sciences de l'Aliment et des Industries Agroalimentaires (ESSAIA), El Harrach, Alger, Algérie; 4 Aix Marseille Univ, IRD, VITROME, IHU Méditerranée Infection, Marseille, France; University of Kentucky College of Medicine, UNITED STATES

## Abstract

**Background:**

Leptospirosis is an important worldwide zoonosis. This disease is caused by pathogenic species of the genus *Leptospira* which are maintained in the environment via chronic renal infection of carrier animals which can be asymptomatic excretors of the organisms in their urines and become a source of infection for humans and other hosts. The prevalence of animal leptospirosis in Algiers, Algeria, is unknown.

**Methodology/principal findings:**

Real-time PCR and standard PCR and sequencing were used to detect pathogenic *Leptospira* organisms in the urines of stray dogs and cats in Algiers. In the presence of appropriate controls, none of the 107 cat urine samples were positive while 5/104 (4.8%) canine urine samples (asymptomatic mixed-breed dogs, three females and two males) were positive in two real-time PCR assays targeting the *rrs* and *hsp* genes. The positivity of these samples was confirmed by partial PCR-sequencing of the *rpo*B gene which yielded 100% sequence similarity with *Leptospira interrogans* reference sequence. In this study, *L*. *interrogans* prevalence was significantly higher in dogs aged < one year (16.46% - 29.41%) than in adults (0%) (P value = 0.0001) and then in the overall dog population (2.68% - 4.8%) (P = 0.0007).

**Conclusions/significance:**

These results suggest that dogs are maintenance hosts for zoonotic leptospirosis in Algiers, Algeria. To face this situation, effective canine vaccination strategies and raising public health awareness are mandatory. Further investigations incorporating a larger sample in more localities will be undertaken to document the epidemiology of urban animal leptospirosis in Algeria at large.

## Introduction

Leptospirosis is a worldwide disease that affects wild and domestic animals and human populations. Affected persons are primarily farmers, fishermen, veterinarians and people working in sewers and slaughterhouses [1]. This zoonosis is caused by pathogenic spirochetes of the genus *Leptospira* which colonize the renal tubules where they reproduce before being excreted via the urines [2]. Infected urines or contaminated water are sources of leptospirosis infection and *Leptospira* can enter the body of mammalian hosts via lacerations in the skin, contacts with mucosa or conjunctiva and inhalation of aerosols [3–5]. Some host animals such as dogs may have an asymptomatic form or may suffer from a wide range of clinical manifestations, including hepatic and renal failure and severe pulmonary hemorrhage [6]. Asymptomatic and chronic carrier dogs can be maintenance hosts [7] acting as sources of infection and therefore cause a public health problem [8]. Formerly, it was thought that domestic cats were resistant to leptospirosis infection and many practitioners did not consider feline leptospirosis in the differential diagnosis of other diseases [9]. However, recently published reports on feline leptospirosis conclude that cats are exposed to *Leptospira* and may play a role in the epidemiology of this disease [10–12].

As a neglected tropical disease, leptospirosis has been increasingly observed in urban settlements, especially in slums in developing countries [6]. The prevalence of animal leptospirosis in Algiers, Algeria, is unknown. Only two studies were published about human leptospirosis in Algeria. These two serological investigations were conducted on patients of the Tizi-ouzou Hospital. The first one reported 48 cases of leptospirosis from 2006 to 2007 and the serogroup icterohaemorrhagiae was identified in 60% of cases [13]. In the second prospective study, 175 positive patients were diagnosed from 2005 to 2008, among the serovars identified, icterohaemorrhagiae and grippotyphosa were predominant [14].

The aim of the present work was to detect pathogenic *Leptospira* organisms in the urines of stray dogs and cats in Algiers.

## Methods

### Ethic statement

The study was submitted to and approved by the ethics committee and decision board (number 416/2017) of EPIC- H.U.P.E (EPIC: Entreprise publique à caractère industriel et commercial; H.U.P.E: Hygiène Urbaine et Protection de l’environnement) of Wilaya of Algiers (Ex: HURBAL). HURBAL was created in 1994 with a new status: EPIC-H.U.P.E under the register number: 16/00-0013132B00. EPIC- H.U.P.E is an institution affiliated with the Algerian Ministry of the Interior and the Local Government and the Algerian Ministry of Water Resources and Environment. In the context of the National Program for Rabies Control, EPIC- H.U.P.E captures stray dogs and cats in Algiers. Once captured, stray animals were housed in cages and euthanized after expiration of the seven day legal waiting time (in order to allow for owners to claim their pets), in compliance with the Algerian legislation for the protection of animals (Law 01/04/1994), which our protocol respected.

### Study design and sampling

This study was designed to screen for the presence of *Leptospira* spp. organisms in stray cats and dogs in the region of Algiers, in the absence of any data available on that topic. Therefore, in this study, we aimed at collecting only the urines of the animals for the molecular detection of *Leptospira* spp. DNA. Urine specimens were aseptically collected between April 2017 and November 2017 were via cystocentesis from 211 stray animals (104 dogs and 107 cats). These animals were captured in the 57 municipalities of the region of Algiers. The sampling was realized in animal shelters with an average of seven animals sampled per week. The age of each animal was estimated, based on dentition and physical aspect. Information concerning sex, breed and clinical status was noted. Samples were stored at -20°C before being transported to the IHU Méditerranée Infection, Marseille, France, for PCR testing and culture was not performed. Up to 3 mL of each urine sample was centrifuged at 15,000 g for 20 minutes [15], the supernatant was discarded and the pellet was suspended in 200 μL of sterile phosphate-buffered saline solution (PBS, pH 7.2) [16].

### DNA extraction

A total of 200 μL of DNA was extracted using the QIAamp Tissue Kit by QUIAGEN-BioRobot EZ1, according to the manufacturer’s instructions (Qiagen, Hilden, Germany). Extracted DNA was stored at -20°C under sterile conditions until used in PCR assays.

### Real time PCR

Extracted DNA was used in qPCR amplifications to detect pathogenic *Leptospira* organisms. The final qPCR reaction mixture consisted of 5 μL DNA with 15 μL of mix from the Roche PCR Kit (Roche Diagnostics, Meylan, France). The components of the final reaction mixture of these PCR assays are given in Table 1. A homemade plasmid containing sequences specific to *Leptospira* spp. was used as a positive control. Three negative controls were incorporated into each PCR run. Results were recorded as positive when the cycle threshold (Ct) was lower than 33. We performed real-time PCR (qPCR) with two systems (Table 2) in order to confirm the positivity of the samples according to current standards in microbiology. The first system targets a 88-pb fragment of the *rrs* gene coding for 16S rRNA of pathogenic *Leptospira*: 16S rRNA Forward (5’-CCCGCGTCCGATTAG-3’), 16S rRNA Reverse (5’-TCCATTGTGGCCGRACAC-3’) and 16S rRNA Probe (5’-CTCACCAAGGCGACGTCGGTAGC-3’) were analyzed as previously described [17]. The second system targets a 103-pb fragment of the *hsp* gene of *L*. *interrogans*: Lint_hsp_MB Forward (5’-CCCGCGTCCGATTAG-3’), Lint_hsp_MB Reverse (5’-TCCATTGTGGCCGRACAC-3’) and Lint_hsp_MB Probe (5’-CTCACCAAGGCGACGTCGGTAGC-3’) were analyzed as previously described [18]. The PCR cycling parameters for the qPCR were 5 min at 95°C followed by 39 cycles each consisting of 5 sec of denaturation at 95°C and 30 sec of annealing at 60°C.

**Table 1:** Concentration of components in the final reaction mixtures of the real-time polymerase chain reaction (qPCR) assays used in this study.

Reagent	16S rRNA qPCR (1X)	Hsp qPCR (1X)
Mix Roche (LightCycler® 480 Probes Master)	10 μL	10 μL
Water volume	3 μL	3 μL
Forward primer	0.5 μL	0.5 μL
Reverse primer	0.5 μL	0.5 μL
Probe	0.5 μL	0.5 μL
Uracyl DNA Glycosidase (UDG)	0.5 μL	0.5 μL

**Table 2:** Primers and probes used in this study.

PCR assay	Primer and probe sequences	References
16S rRNA	Forward primer: (5'-CCCGCGTCCGATTAG-3') Reverse primer: (5’-TCCATTGTGGCCGRA/GACAC-3') Prob: (5'-CTCACCAAGGCGACGATCGGTAGC-3')	[17]
Lint_hsp_MB	Forward primer: (5’-CCCGCGTCCGATTAG-3’) Reverse primer: (5’-TCCATTGTGGCCGRACAC-3’) Prob: (5’-CTCACCAAGGCGACGTCGGTAGC-3’)	[18]
*rpoB*	Forward primer: (5’-CCTCATGGGTTCCAACATGCA-3’) Reverse primer: (5’-CGCATCCTCRAAGTTGTAWCCTT-3’)	[19]

### Standard PCR and sequencing

Samples that tested positive by qPCR were confirmed by standard PCR and sequencing in order to achieve a 100% specificity. The final standard PCR reaction mixture consisted of 5 μL of DNA with 15 μL of mix from the Roche PCR Kit (Roche Diagnostics). The components of the final reaction mixture of these PCR assays are given in Table 3. Samples were then confirmed by standard PCR using primers which amplified a 592-pb fragment of the *rpoB* gene: Lept 1900 Forward (5’-CCTCATGGGTTCCAACATGCA-3’) and Lept 2500 Reverse (5’-CGCATCCTCRAAGTTGTAWCCTT-3’), as described by La Scola et al., 2006 [19] (Table 2). The PCR cycling parameters for the standard PCR were 15 min at 95°C followed by 35 cycles of each consisting of 30 sec denaturation at 95°C, 30 sec annealing at 51°C and 6 min extension at 72°C in an ABI Thermocycler (Applied Biosystems Gene Amp PCR System 2700, Villebon sur Yvette, France). Negative controls were incorporated into each PCR run.

**Table 3:** Concentration of components in the final reaction mixtures of the standard polymerase chain reaction assay used in this study.

Reagent	*rpoB* Standard PCR (1X)
Ampli Taq Master Mix	12.5 μL
Water volume	6 μL
Forward primer	0.75 μL
Reverse primer	0.75 μL

The amplified PCR products were separated via gel electrophoresis using a 1.5% agarose gel stained with Sayber Safe (ThermoFisher, Paris, France). The DNA bands were visualized and photographed under ultraviolet light. PCR products were purified and sequenced with *rpoB* primers as described previously [19]. All obtained sequences were assembled and edited using ChromasPro (version 1.7.7). The sequences were then analyzed by Basic Local Alignment Search Tool (BLAST) and compared with sequences available in the GenBank database.

### Statistical analyses

Statistical analyses were done by MEDCALC® online software https://www.medcalc.org/calc/comparison_of_proportions.php using the “N-1” Chi-squared test as recommended by Campbell., 2007 [20] and Richardson., 2011 [21]. The confidence interval was calculated according to the recommended method given by Altman et al., 2000 [22].

## Results

### Sample collection

From April 2017 to November 2017, a total of 104 stray dogs and 107 stray cats captured in Algiers, Algeria were sampled. These animals lived in urban areas, spending most of their time exclusively outdoors and did not receive any vaccine. Of the 104 dogs, 69/104 (66.34%) were males and 35/104 (33.65%) were females. The canine population consisted predominantly of mixed-breed dogs; other dogs belonged to the following races: German shepherd, American Staffordshire, shepherd crosses and Pit-bull. The dogs’ age ranged between 2 months and 11 years. Among the 107 cats, 66/107 (61.68%) were males and 41/107 (38.31%) were females. The cats were described as mostly belonging to mixed breeds, some belonging to European or Siamese crossbreeds. The 107 cats sampled were estimated to be under 5 years of age. All sampled animals were apparently healthy.

### Real time PCR

qPCR targeting the 16S rRNA gene of pathogenic *Leptospira* and the hsp gene of *L*. *interrogans* revealed that none of the 107 urine samples of cats tested were positive while 5/104 (4.8%) dogs were positive. These five urine specimens were positive in the two qPCR systems (rrs and hsp). Using the Cts obtained from the 16S rRNA qPCR reactions and a calibration curve previously described for this system [23], we extrapolated the number of leptospira genomes per positive reaction (Table 4). Positive dogs were all very young, under one year of age. Three were females and two were males. All positive animals belonged to mixed-breeds (Table 4). In this study, *L*. *interrogans* prevalence was significantly higher in dogs aged < one year (5/17; 29.41%) than in adults (0/87; 0%) (P value = 0.0001, 95% CI: 12.73 to 53.13) and than in the overall dog population (5/104; 4.8%) (P = 0.0007, CI: 7.1929 to 48.2975). The sensitivity of our screening test based on the detection of 16S rRNA using qPCR was previously estimated to be of 56% [24], accordingly, the prevalence rate obtained here was estimated to be of 2.68%-4.8%. There was no significant difference regarding prevalence between males (3/69, 2.43% - 4.34%) and females (2/35, 3.19% - 5.71%) (p = 0.758).

**Table 4:** Information relative to animals detected positive for *L*. *interrogans* DNA in urine samples: Age, sex, race, number of genomes per positive pPCR reaction. (The values were extrapolated from the calibration curve (21) using the Ct obtained from the 16S rRNA system).

Case (N°)	Age	Sex	Race	Ct rRNA (Log *Leptospira* genome/reaction)
1 (06)	4 months	M	Mixed-breed	26.6 (9.5×10^3^)
2 (17)	7 months	M	Mixed-breed	23.71 (9.6×10^4^)
3 (34)	4 months	F	Mixed-breed	23.47 (9.3×10^4^)
4 (40)	5 months	F	Mixed-breed	25.26 (1.02×10^4^)
5 (87)	4 months	F	Mixed-breed	17.54 (1.05×10^6^)

M = male; F = female.

### Standard PCR and sequencing

All five urine samples detected positive by real-time PCR were confirmed with gel-based PCR assay targeting the *rpoB* and subjected to sequencing analysis. The BLAST (www.ncbi.nlm.nih.gov/blast) analysis of the *rpoB* gene sequence from all samples, once compared with sequences available in the GenBank database, confirmed *Leptospira* infection. The BLAST analysis yielded a 100% sequence homology with *L*. *interrogans* homologous gene fragment (GenBank accession no. CP020414.1).

## Discussion

In Algeria, the exact morbidity and the mortality due to leptospirosis are unknown. In 1975, a study based on serology reported seven cases in a military group in Algiers’ suburbs [25]. Two more recent studies reported cases of leptospirosis among hospitalized patients in the region of Tizi-Ouzou, located 100 km east of the capital Algiers. The investigation of 48 patients from 2006 to 2007 in the rural area of Tala-Athmane revealed that they were living in close contact to two garbage dumps invaded by rodents, the cases were confirmed serologically by the microagglutination test (MAT) and more than 60% (n = 29) were from the serogroup icterohaemorrhagiae [13]. A second prospective study conducted from 2005 to 2008 in the same region reported 173 cases among hospitalized patients, the cases were confirmed serologically with a predominance the of serovars icterohaemorrhagiae and grippotyphosa [14].

However, the prevalence of this zoonosis in reservoirs is totally unknown in Algeria as the only report of it is the observation of *Leptospira* organisms in histological sections of the liver in dogs presenting with severe jaundice, subcutaneous hemorrhages and acute nephritis [26]. For this pioneering study of animal leptospirosis in Algeria, we used urines in which leptospiral DNA can be found much longer than in blood [27–29]. The need for a rapid diagnosis of leptospirosis has led to the development of numerous PCR assays, which appeared to have more applicability in determining zoonotic risks [30]. This method is rapid, sensitive, specific and robust and many PCR assays were developed to detect universal *Leptospira* genes such as *gyr*B, *rrs* and *sec*Y genes or genes restricted to pathogenic species such as *lip*L32, *lfb*1, *lig*A and *lig*B2 [5]. In this study we aimed at retrospectively confirming the positive detection of *Leptospira* spp. DNA by targeting two different molecular targets. We chose the rrs and hsp as genus-level targets of identification and *rpo*B sequencing for the identification at the species level of pathogenic leptospira.

We confirmed the presence of *L*. *interrogans* in the urines of stray dogs in Algiers using qPCR and standard PCR-sequencing targeting universal and pathogen-related genes. We observed an overall prevalence of 2.68% - 4.8% and a high prevalence of 29.41% in the specific population of young dogs aged < one year. All animals were apparently healthy, indicating asymptomatic carriers. These dogs were always outside, in contact with garbage and small rodents which were likely sources of infection. Crowding animals in unsanitary quarters is associated with a high prevalence of infection since animals may acquire the disease through contact with urines from infected dogs or infected rodents [31]. Despite the fact that we did not attempt to isolate *Leptospira* spp. to confirm the role of stray dogs as reservoirs, present data indicate that stray dogs would indeed be good sentinels to know which serovars/groups are circulating in the rodent populations.

**Fig 1:**
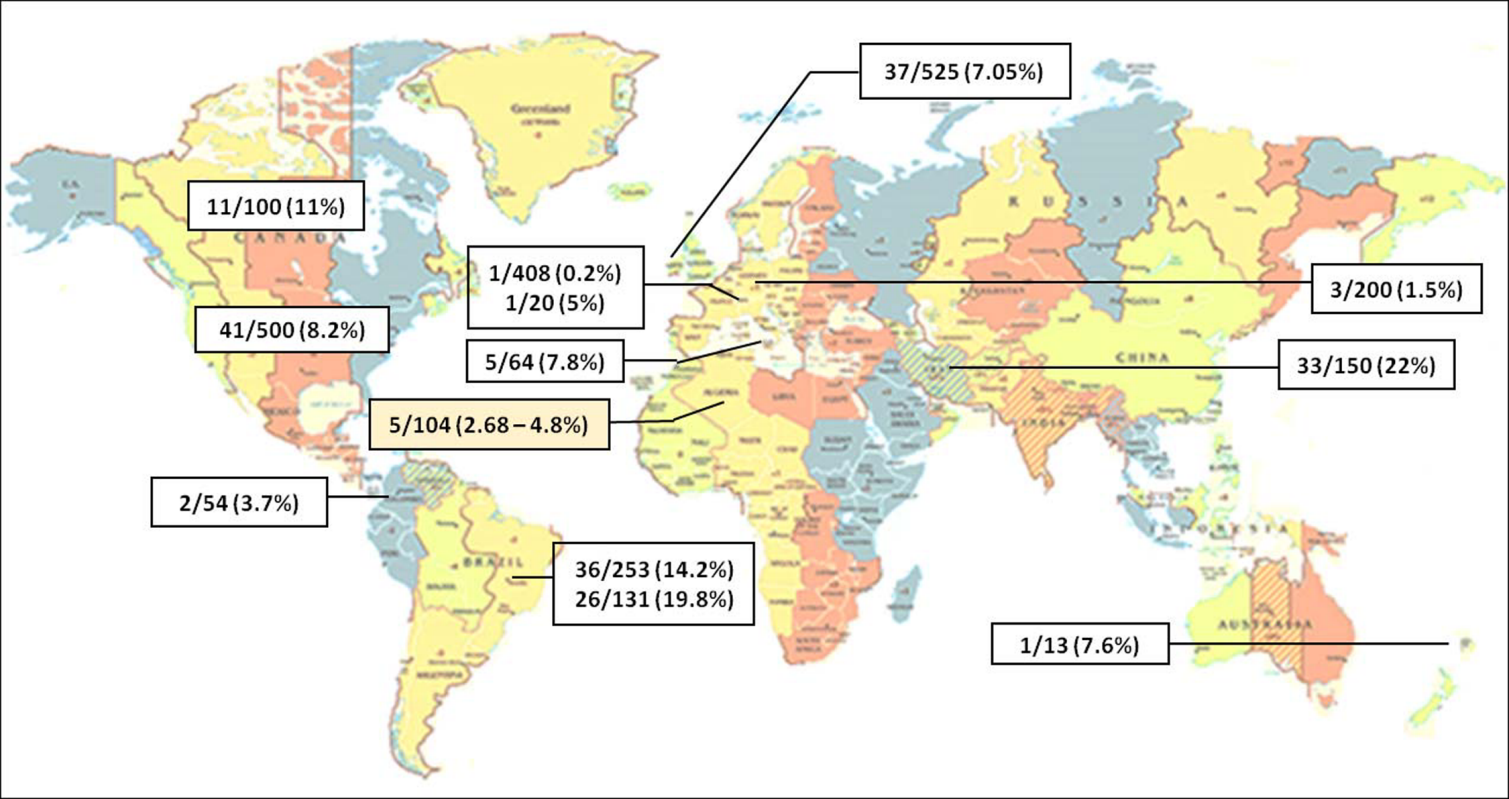
Molecular detection of *Leptospira* spp. DNA in the urines of dogs, worldwide. Germany, 3/200 (1.5%) [8], the USA, 41/500 (8.2%) [14], Teheran (Iran), 33/150 (22%) [34], Canada, 11% (11/100) [35], Sicily (Italy), 5/64 (7.8%) [36], Ireland, 37/525 (7.05%) [37], Porto Alegre City (Brazil), 36/253 (14.2%) [38], Switzerland, 1/20 (5%) [39], Colombia 2/54 (3.7%)[40], New Caledonia, 1/13 (7.6%) [41], Brazil, 26/131 (19.8%) [42], Switzerland, 1/408 (0.2%) [43], Algiers (Algeria), 5/104 (2.68% - 4.8%) [Present work].

Subclinical, latent leptospirosis in dogs has regularly been reported and can also be observed in unsteady vaccinated animals [32]. In addition, there are data suggesting that clinically asymptomatic dogs can be chronic carriers, shedding *Leptospira* via urines into the environment [30, 33, 34]. Many epidemiological studies were conducted worldwide on the renal carriage of leptospirosis in dogs using molecular tools [8, 14, 34–43], showing a prevalence between 0.2% and 22% worldwide Fig 1). Some other studies have not found the presence of pathogenic *Leptospira* species in dogs in the USA (0/100) and in Egypt (0/25), but only the presence of antibodies as an evidence of the exposure to the disease [44] [45]. This may be due to the minute amount of *Leptospira* DNA in the blood and urine specimens of infected dogs. Therefore, a highly sensitive PCR platform is required to obtain an accurate diagnosis and an innovative approach must be adopted to maximize sample DNA input in the PCR or by increasing the volume of urine for DNA extraction. This could be achieved by high-speed sedimentation of a milliliter volume of urines and performing DNA extraction from the complete sediment, as performed in the present study [46]. Positive dogs in our study were very young dogs (under one year of age). However, it has been shown in the USA that dogs aged between 4 and 6.9 years and between 7 and 10 years faced a significantly higher risk of being infected than dogs under one year of age [47]. In a study conducted in Reunion Island, only adult dogs were positive for leptospirosis [48]. The difference in age range can be explained by the fact that the young stray dogs of our study were not vaccinated against leptospirosis and exposed at an early age to the bacterium in their environment. Furthermore, the canine population of two previous studies [47, 48] was composed of domestic dogs with different risk factors and exposure to other maintenance host species.

In the present work, all sampled cats were qPCR-negative for *L*. *interrogans*. Some studies yielded a seroprevalence between 4% and 30% in different countries including Australia [49], Scotland [50], Greece [51], Iran [52], Spain [53], Canada [54], Taiwan [11], Chile [10], Colombia [55] and Brasil [56]. Some other studies found DNA sequences of *Leptospira* in urine samples indicating a renal carriage of leptospirosis in cats. In Taiwan, DNA of pathogenic *Leptospira* was detected in 67.8% (80/118) of the urine samples of cats including 71 stray cats and nine household cats [11]. In Canada, DNA of *Leptospira* was detected in the urines of sick cats (6/113) and healthy cats (2/125), corresponding to a prevalence of 8/238 (3.3%) [12]. In Germany, urine samples from 7/215 (3.2%) cats were PCR-positive [57]. In Quebec, PCRs on urines detected urinary excretion in 3.2% of the 250 cats sampled [58]. These results suggest that cats may have a role in the transmission of leptospirosis, as a reservoir or as an accidental host. The role of cats in the transmission of leptospirosis should be reevaluated, as it might in fact be underestimated.

In conclusion, this study demonstrates the high number of leptospiral carriers among asymptomatic young dogs. Improving the awareness of dog owners and the prevention of canine leptospirosis could be a valuable asset for human leptospirosis prevention [59]. The prevalence of leptospirosis in countries where dogs are correctly vaccinated remains high, this is mainly due to the difference in epidemiology between the different serovars and the absence of cross-protection in a vaccine, thus, a more effective vaccine needs to be developed [60]. The implementation of a surveillance system for canine leptospirosis, using dogs as sentinels for human risk assessment, could also provide a valuable tool for estimating and in turn minimize the risk for humans [61]. Further studies on leptospirosis in other animals and other regions in Algeria should be considered to clarify the status of this disease in our country. Also, further studies in the region of Algiers and other regions of Algeria will have to determine the serotypes of circulating leptospira in order to refine the epidemiology of leptospirosis in Algeria.
